# The risk of being bitten by a dog is higher on hot, sunny, and smoggy days

**DOI:** 10.1038/s41598-023-35115-6

**Published:** 2023-06-15

**Authors:** Tanujit Dey, Antonella Zanobetti, Clas Linnman

**Affiliations:** 1 Department of Surgery, Center for Surgery and Public Health, Brigham and Women’s Hospital, Harvard Medical School, Boston, MA USA; 2 Department of Environmental Health, Harvard T.H. Chan School of Public Health, Boston, MA USA; 3 Spaulding Neuroimaging Laboratory, Department of PM&R, Spaulding Rehabilitation Hospital, Harvard Medical School, Boston, MA USA

**Keywords:** Ecological epidemiology, Psychology and behaviour, Epidemiology

## Abstract

Humans commit more violent crimes when temperature and air pollution is higher. Here, we investigate if also the day-to-day rates of dogs biting humans is influenced by environmental factors. 69,525 reports of dogs biting humans, sourced from public records on animal control requests and from ER records, were analyzed. The impact of temperature and air pollutants were evaluated with a zero-inflated Poisson generalized additive model, while controlling for regional and calendar effects. Exposure–response curves were used to assess the association between outcome and major exposure variables. We find that the rates of dogs biting humans increases with increasing temperature and ozone, but not PM_2.5_ exposure. We also observed that higher UV irradiation levels were related to higher rats of dog bites. We conclude that dogs, or the interactions between humans and dogs, are more hostile on hot, sunny, and smoggy days, indicating that the societal burden of extreme heat and air pollution also includes the costs of animal aggression.

## Introduction

Aggression is a common behavior across species, with sometimes adaptive advantages to defending a territory, obtaining limited resources, competing for mates, or protecting members of the pack or tribe. Many acts of aggression may be conceptualized as the result of an imbalance between prefrontal “top-down” control systems and hyper-responsivity of limbic regions triggered by anger provoking stimuli^1^, a circuit that further appears modulated by striatal encoding of reward^2–4^. Human aggression has complex psychological and sociological roots, yet some external factors increase aggression across species: Higher temperature increases the likelihood of aggression among humans^5–7^, Rhesus monkeys^8^, rats^9^ and mice^10^. Inter-species aggression—dogs biting humans—has also been linked to higher temperatures^11^.

Short term exposure to air pollutants (particulate matter < 2.5 μm (PM_2.5_) and ozone)^12–18^ also appears to increase the incidence of human violent crime, as based on time-series analyses of air quality and criminal records data. It is not known if the link between air pollutants and aggression extends to other species.

To further investigate the link between air pollution exposure and aggression, we here explore public record of dogs biting humans. Dog bites represent 0.3% of all emergency department visit, and are a source of cosmetic disfigurement, trauma, finger amputation and occasional severe craniofacial injury and fatality^19,20^.

Multiple risk factors for dog bites have been identified, including dog specific factors (sex, castration/spay status, breed), victim factors (age, gender, familiarity with dog, victim behavior), and dog-victim interactions^21–25^.

The goal of this study was to determine potential environmental contributions to the daily prevalence of dog bites in 8 US cities during the years 2009 to 2018 in relation to temperature, the air pollutants PM_2.5_ and ozone, while controlling for precipitation, UV irradiation, calendar, and seasonal factors.

## Methods

Dog bite incidents, typically recorded by city animal control authorities, were obtained from publicly available repositories for, Dallas^26^ and Houston^27^ (Texas), Baltimore (Maryland)^28^, Baton Rouge (Louisiana)^29^, Chicago (Illinois)^30^, Louisville^31^ (Kentucky) and New York City^32^ (New York). Data on dog bite incidents in Los Angeles^23^ (California) were compiled by Dr. Lisa Smith (Los Angeles County Department of Public Health) and Dr. Tony Kuo (University of California, Los Angeles) and used with permission. The above sources were selected because of availability and coverage, i.e., covering daily incidence over several years. As the included cities are of different size and used different reporting methods, we used the relative daily incidence in each city (daily incidence/city average daily incidence) as the outcome variable.

Daily counts of dog bites were zero-inflated (i.e., many days without incidents, Fig. S1 in the Appendix) and data was modeled utilizing a Poisson distribution in the ZIGAM model.

We obtained daily 24-h averages of PM_2.5_ (μg/m^3^) and daily 8-h maximum ozone (ppm) from the Environmental Protection Agency’s Air Quality System^33^ from all monitors within city limits. Average levels across all monitors were calculated for each city. We sourced precipitation and maximum daily temperatures from the National Oceanographic and Atmospheric Administration’s Climatology Network^34^. We sourced daily UV index for available cities from the National Weather Service Climate Prediction Center^35^. We excluded PM_2.5_ values more than 35 μg/m^3^ as there were few (0.12%) observations beyond this value.

To account for homogeneity of exposure effects, we standardized values of PM_2.5_, ozone, daily maximum temperature, precipitation, and UV index.

To estimate the association between day-to-day variations in exposure (PM_2.5_, ozone and temperature) on dog bite rates we applied a zero-inflated Poisson generalized additive (ZIGAM) model. We applied this model given that the daily counts of dog bites had many days without incidents (see Fig. S1 in the Appendix) and were therefore zero-inflated.

To adjust for potential confounding by seasonality and long term trend we included in the model penalized cubic splines of date per year of data. We also adjusted for federal holidays and weekends, and for cities with a categorical variable. The models included simultaneously daily PM_2.5_, ozone, maximum temperature, precipitation, and UV index. We ran multivariable models in a way where we have used date as non-linear term in the model and all the covariate as linear term to the model to accomplish the goal of create the exposure–response curve for each of the pollutants, separately. The exposure–response function (ERF) for each pollutant and temperature, in separate models, were calculated using a bootstrapping procedure: First we sample the data with replacement from the original data set. Then we apply the ZIGAM model on the bootstrapped data to build the model. Second, we then predict on the original data set at a given fixed value of the exposure variable, for example, PM_2.5_ on the range between the minimum and maximum value in the data set and averaged all predicted values to get an estimate of the ERF at that fixed value. Finally, we repeat Steps 1–2, for a large amount of time, to get all averaged ERF values at those given exposure levels. This way we get a bootstrapped version of the prediction model which considers the uncertainty of the original data and then since we are predicting on the original dataset, the distribution of covariates is set to be the same as in the original dataset. This approach allows us to estimate the ERF and also to compute the corresponding 95% confidence interval.

Initial graphical assessment on dog bites rates displayed some seasonality during the winter months in contrast to the non-winter months, as do other exposures. To examine whether the effects varied by winter and non-winter months we did stratified analysis by winter and non-winter months. In sensitivity analysis we examined whether the effect of ozone was confounded by UV index by excluding this variable from the model.

All hypothesis tests were two-sided and p values < 0.05 were considered statistically significant.

All analysis was performed using R software version 4.1.0 (R Foundation for Statistical Computing, Vienna, Austria (2020)). Main implementation of the ZIGAM model was run using the gam function of the mgcv R package.

## Results

**Figure 1:**
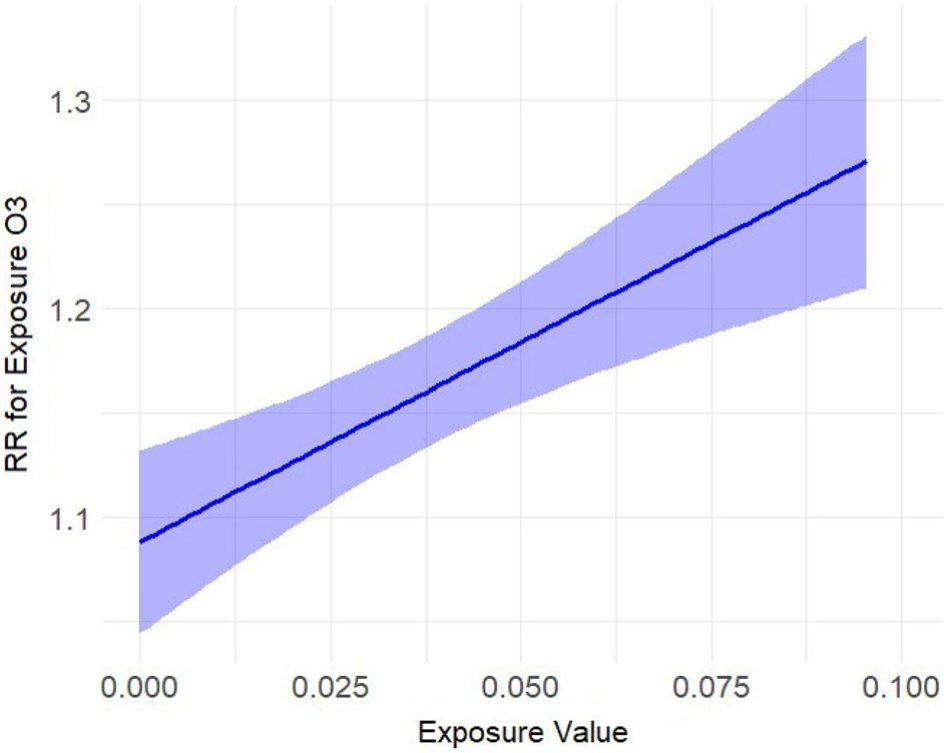
Estimated exposure–response curve for the exposure ozone (in ppm) on the rate of dog bites.

**Figure 2:**
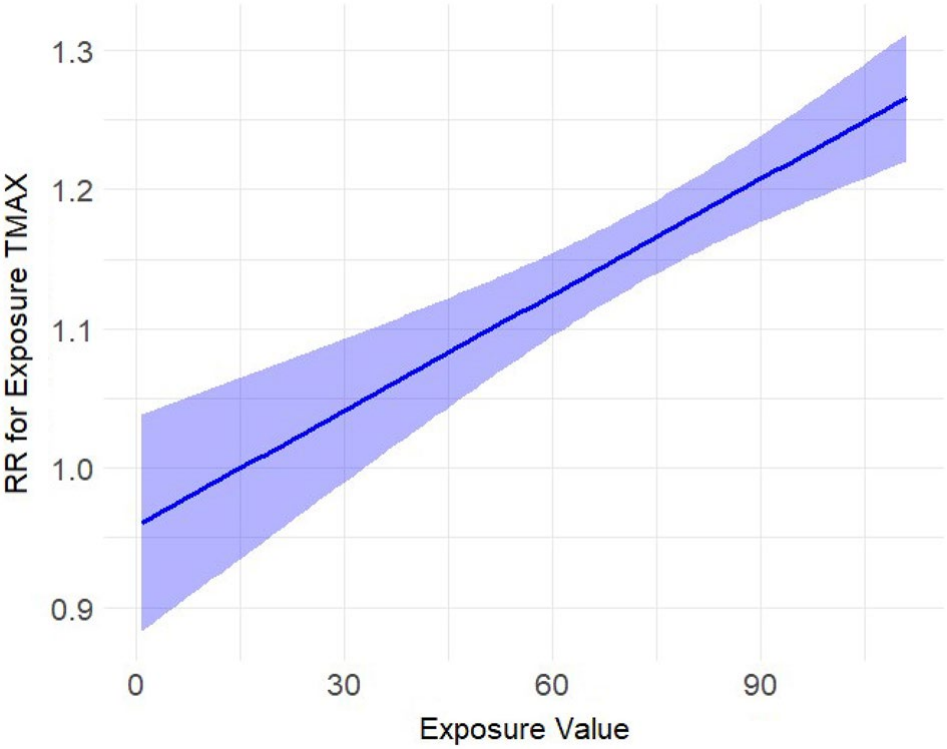
Estimated exposure–response curve for temperature (in Fahrenheit) on the rate of dog bites.

**Figure 3:**
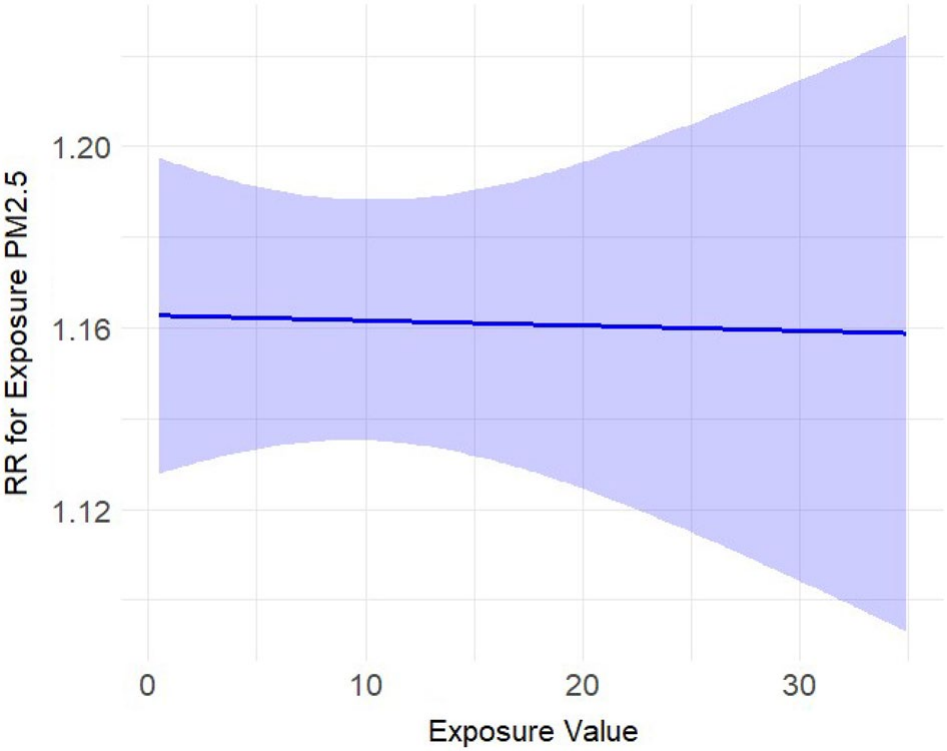
Estimated exposure–response curve for the exposure PM_2.5_ (in μg/m^3^) on the rate of dog bites.

We included 11,082 complete datapoints, with a total of 69,525 reported dog bite incidents and an average three dog bites per day (Interquartile range (IQR) (one to eight incidents, see Supplement Table 1)), across 8 cities spanning 10 years. We find that dog bite incidence increased with increasing ozone (Fig. 1), temperature (Fig. 2), and UV irradiation, and decreased on rainy days and on holidays (see Table 1). There were however no effects of PM_2.5_ (Fig. 3). Results for ozone and UV irradiation remained significant when analyzing winter and non-winter months separately (Supplemental Table 3). As ozone levels covary with temperature and UV irradiation, in sensitivity analyses (see Supplement) we further modeled the results excluding either UV or ozone and found that the estimates of other variables were not influenced by either of them (see Supplement Table 2).

**Table 1:** Results from the Zero-inflated Poisson generalized additive model.

Characteristic	IRR	95% CI^a^	p-value
Ozone	1.03	1.02, 1.04	** < 0.001**
PM_2.5_	1.00	0.99, 1.01	0.9
Precipitation	0.99	0.98, 1.00	0.031
Temperature (Max)	1.04	1.03, 1.06	** < 0.001**
UV	1.11	1.09, 1.13	** < 0.001**
Holidays and weekends	0.94	0.90, 0.99	**0.015**
Winter months	1.04	1.02, 1.07	**0.002**

Significant values are in bold.

This model was also adjusted for 8 cities (as a categorical variable) as a linear effect and dates across the years was adjusted as a penalized cubic spline (with adaptive knots) in the model.

^a^CI: Confidence Interval.

## Discussion

Our results indicate that the daily incidence of dog bites is influenced by multiple environmental variables, including ozone, temperature, precipitation, and UV levels, but not PM_2.5_. Sensitivity analyses indicated that these relationships were stable and not greatly influenced by models or co-variance between variables. This is in line with prior studies on the impact of ozone on human aggression^13,15,17,18^, and studies on human aggression and temperature^36^. The effect of UV irradiation increasing aggression is in line with recent studies indicating increased aggression and increased sex-steroid levels after UVB exposure in mice and men^37^.

Ozone has a strong smell, is highly reactive and triggers oxidative stress in the airways and impairs pulmonary function. Due to its reactivity, ozone is not thought to penetrate beyond the membranes lining the respiratory tract and lungs, so behavioral effects may occur via generation of free radicals from lipid peroxidation. In humans, ozone exposure triggers the release of multiple messenger pathways, including serum amyloid A^38^, interleukin-6^39,40^ and interleukin-8^41^ and activation of the hypothalamic pituitary adrenal (HPA) axis^42^. Behavior may thus be influenced by a general stress response to pollutants triggered by lung inflammatory messengers. More direct effects on brain function are also possible: In rats, acute ozone exposure rapidly increases dopamine^43^, noradrenaline, dihydroxyphenylacetic acid, and 5-hydroxyindolacetic acid in the striatum and midbrain^44^. Ozone exposure further stimulates catecholamine biosynthesis in the hindbrain noradrenergic A2 group, catecholamine turnover is increased in the cortex, but decreased in the striatum^45^. In human experimental ozone exposure studies, 4 h of 200 ppb ozone exposure led to a 79% increase in 8-isoprostane (8-ISO), a measure of lipid oxidation, 18 h after exposure^46^. Notably, 8-ISO levels are elevated in intermittent explosive disorder, and further correlated to measures of actual aggressive behaviors^47^. As the neural circuitry for aggressive behaviors is conserved across mammals and given the impact of ozone on basal ganglia dopaminergic function, we speculate that ozone may influence aggressive behavior via impacts on dopamine turnover in the striatum. While combustion derived PM_2.5_ has been detected in the brains of both dogs^48^ and humans^49^, we did not observe and effect of PM_2.5_ on dog bite incidence. Compared to humans, dogs have a much larger surface area of olfactory epithelium, more olfactory receptors, and a larger olfactory bulb^50,51^. As such, anatomical differences between humans and dogs may account for the lack of effect in this study.

We utilized animal control and hospital records to evaluate the impact of temperature and air pollutants on dog bite incidence. However, survey data indicates that the true burden of dog bites is much higher than reported in hospital data^52^ and only a small percentage of dog bites require extensive medical treatment or hospitalization^53^. Our results are therefore likely indicative of more severe dog bite incidents. According to prior studies, most dog bites arise from a dog known to the victim, and most bites are related to interacting or attempting to interact with the dog^21,25^. While it is likely that human–dog interactions increase on days with higher temperature and higher UV irradiation (i.e., sunny days), our analysis indicates that ozone levels further contribute to the risk of dog bites, an effect present in both winter and summer months independently. Moreover, our analysis indicates a slight decreased risk on weekends and holidays, suggesting that ample time for dog–human interactions does not increase risk.

A limitation of our analysis is that public records of dog bites do not provide more detailed information about dog breed, sex, castration/spaying status, nor for bite severity, victim age, gender, familiarity with dog and the interactions leading up to the dog bite, all factors that impact the risk and consequence of dog bites^21,23–25^.

We included data spanning 2009 to 2019. Earlier datapoints were not publicly available from our sources. We did not include data from the COVID-19 era. During COVID-19 lockdowns, air pollution decreased, but pediatric emergency department visits for dog bites increased^54,55^. This suggest that other factors, such as forced proximity, may be a larger determinant in dog-on-human aggression. According to the American Veterinary Medicine Association, dogs bite primarily as a reaction to something, such as stressful situations, a scare, startle, or threat, or to protect food, toys or their puppies^56^. Dogs might bite defensively or to be left alone^22,24,25^. In our analysis, it is unclear if dog behavior is directly altered by ozone and heat, or, if the observed increase in dog bites is a consequence of altered behavior imposed by the human victim and/or the dogs master, which in many cases are the same individual^21^.

The effects of increasing temperature and air pollutants on *human* aggression, as indexed by police records, are well established^5–7,12–17^. Yet police records of criminal activity, while extensive and well documented, may have systematic biases: less than 45% of violent crimes are reported to law enforcement^57^. Criminal reporting may further be impacted by the behavior of victims and bystanders, as well as by the priorities and resources of law enforcement. The present findings, expand the association between temperature, air pollutants and aggression across species to also include dogs. It is notable that in rodents, exposure to ozone, heat stress, and their combination induces cognitive decline and neuroinflammation^58^. The link between ozone and *aggression* awaits verification such as by randomized double blinded exposure experiments in animals or possibly humans. While cardiovascular and pulmonary health effects of pollution are well established, the present results emphases the impacts on behavior and mental health. Through such mechanism, air pollutants and extreme heat could contribute to higher societal and individual burdens then currently appreciated.

## Supplementary Information

Supplementary Information.

## Data Availability

All data was obtained from public repositories as referenced, except dog bite incidents in Los Angeles^23^ (California) which were compiled by Dr. Lisa Smith (Los Angeles County Department of Public Health) and Dr. Tony Kuo (University of California, Los Angeles) and used with permission. The curated datasets generated and analyzed during the current study are available from the corresponding author on reasonable request, with data from Los Angeles also contingent on original author permission^23^.
