# Author Correction: The risk of being bitten by a dog is higher on hot, sunny, and smoggy days

**DOI:** 10.1038/s41598-023-37841-3

**Published:** 2023-07-03

**Authors:** Tanujit Dey, Antonella Zanobetti, Clas Linnman

**Affiliations:** 1 Department of Surgery, Center for Surgery and Public Health, Brigham and Women’s Hospital, Harvard Medical School, Boston, MA USA; 2 Department of Environmental Health, Harvard T.H. Chan School of Public Health, Boston, MA USA; 3 Spaulding Neuroimaging Laboratory, Department of PM&R, Spaulding Rehabilitation Hospital, Harvard Medical School, Boston, MA USA

Correction to: *Scientific Reports* 10.1038/s41598-023-35115-6, published online 15 June 2023

The original version of this Article contained an error in Affiliation 2, which was incorrectly given as

‘Department of Environmental Health, Harvard School of Public Health, Boston, MA, USA.’

The correct affiliation is listed below:

‘Department of Environmental Health, Harvard T.H. Chan School of Public Health, Boston, MA, USA.’

The original Article has been corrected.

