# Supplementary material for: The risk of being bitten by a dog is higher on hot, sunny, and smoggy days
Source: Sci Rep. 2023 Jun 15;13:8749. doi: 10.1038/s41598-023-35115-6 (PMC10272239; doi:10.1038/s41598-023-35115-6)
Supplement: Supplementary file 1 — Supplementary Information. [file 41598_2023_35115_MOESM1_ESM.docx]

Supplemental material


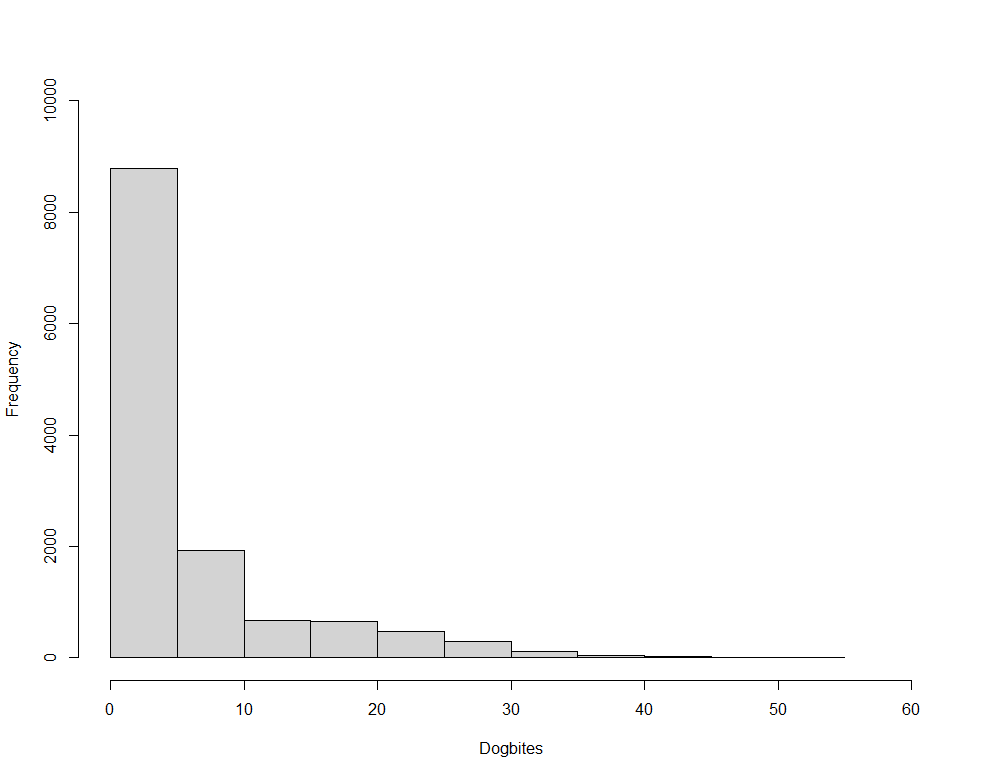


Figure S5: Distribution of the Dog bites based on the entire data set.

Supplement Table 1: Summary statistics of the data set including 11,082 daily observations and a total of 69,525 dog bite incidents

| Characteristic | N = 11,082^a^ |
| --- | --- |
| DOGBITES (daily) | 3 (1, 8) |
| O_3_ | 0.037 (0.028, 0.047) |
| PM25 | 8.8 (6.5, 12.0) |
| Precipitation (mm) | 0.00 (0.00, 0.03) |
| Maximum temperature | 76 (64, 86) |
| UV index | 6.14 (3.33, 8.86) |
| Missing data ^b^ | 219 |
| Holidays | 366 (3.3%) |
| City |  |
| Baltimore | 1,046 (9.4%) |
| Baton | 2,327 (21%) |
| Chicago | 1,094 (9.9%) |
| Dallas | 720 (6.5%) |
| Houston | 1,747 (16%) |
| LA | 1,087 (9.8%) |
| Louisville | 1,968 (18%) |
| NYC | 1,093 (9.9%) |
| Winter month |  |
| No | 5,017 (45%) |
| Yes | 6,065 (55%) |
| ^a^ Median (IQR), ^b^ Missing pollution or meteorological data | |

Sensitivity Analysis:

Supplement Table 2: Results from the Zero-inflated Poisson generalized additive model. Here were interested to investigate whether the estimates of other variables being controlled by the interplay between the effect of UV and Ozone. Therefore, we have run two model excluding either UV or ozone. These two models were also adjusted for 8 cities (as a categorical variable) as a linear effect and dates across the years was adjusted as a penalized cubic spline (with adaptive knots) in the model.

| Characteristic | Model without Ozone | | | Model without UV | | |
| --- | --- | --- | --- | --- | --- | --- |
|  | IRR | 95% CI^a^ | p-value | IRR | 95% CI^a^ | p-value |
| PM25 | 1.00 | 0.99, 1.01 | 0.5 | 1.00 | 0.99, 1.01 | 0.9 |
| Precipitation | 0.99 | 0.98, 1.00 | 0.009 | 0.98 | 0.97, 0.99 | **<0.001** |
| Temperature (Max) | 1.05 | 1.04, 1.06 | <0.001 | 1.07 | 1.06, 1.09 | **<0.001** |
| UV | 1.13 | 1.11, 1.14 | <0.001 |  |  |  |
| O3 |  |  |  | 1.06 | 1.05, 1.07 | **<0.001** |
| Holidays and weekends | 0.95 | 0.94, 0.97 | <0.001 | 0.94 | 0.93, 0.96 | **<0.001** |
| Winter months | 1.05 | 1.02, 1.07 | <0.001 | 0.93 | 0.91, 0.95 | **<0.001** |
| ^a^CI = Confidence Interval | | | | | | |

Supplemental Table 3: Comparison between the whole season model to two other models. Data set was divided into two parts: winter months and no winter months. Here we want to see whether the relationship between the outcome and ozone and PM25 and are changing depending on the seasonal differences. These two models were also adjusted for 8 cities (as a categorical variable) as a linear effect and dates across the years was adjusted as a penalized cubic spline (with adaptive knots) in the model.

| Characteristic | Model for the Winter Months | | | Model for not Winter Months | | |
| --- | --- | --- | --- | --- | --- | --- |
|  | IRR | 95% CI^a^ | p-value | IRR | 95% CI^a^ | p-value |
| Ozone | 1.03 | 1.01, 1.05 | <0.001 | 1.01 | 1.00, 1.03 | **0.033** |
| PM2.5 | 1.01 | 1.00, 1.02 | 0.13 | 1.01 | 1.00, 1.03 | **0.033** |
| Precipitation | 0.99 | 0.97, 1.00 | 0.051 | 0.99 | 0.97, 1.00 | 0.11 |
| Temperature (Max) | 1.06 | 1.05, 1.08 | <0.001 | 0.97 | 0.94, 1.00 | 0.054 |
| UV | 1.12 | 1.09, 1.14 | <0.001 | 1.10 | 1.07, 1.12 | **<0.001** |
| Holidays and weekends |  |  |  |  |  |  |
| 0 | — | — |  | — | — |  |
| 1 | 0.95 | 0.93, 0.97 | <0.001 | 0.94 | 0.92, 0.97 | **<0.001** |
| ^a^ CI = Confidence Interval | | | | | | |
